# Increased Na^+^/Ca^2+^ Exchanger Expression/Activity Constitutes a Point of Inflection in the Progression to Heart Failure of Hypertensive Rats

**DOI:** 10.1371/journal.pone.0096400

**Published:** 2014-04-29

**Authors:** Jesica S. Rodriguez, J. Omar Velez Rueda, Margarita Salas, Romina Becerra, Mariano N. Di Carlo, Matilde Said, Leticia Vittone, Gustavo Rinaldi, Enrique L. Portiansky, Cecilia Mundiña-Weilenmann, Julieta Palomeque, Alicia Mattiazzi

**Affiliations:** 1 Centro de Investigaciones Cardiovasculares, CONICET-La Plata, Facultad de Medicina, Universidad Nacional de La Plata, La Plata, Argentina; 2 Laboratorio de Análisis de Imágenes, Facultad de Ciencias Veterinarias, Universidad Nacional de La Plata, La Plata, Argentina; The University of Manchester, United Kingdom

## Abstract

Spontaneously hypertensive rat (SHR) constitutes a genetic model widely used to study the natural evolution of hypertensive heart disease. Ca^2+^-handling alterations are known to occur in SHR. However, the putative modifications of Ca^2+^-handling proteins during the progression to heart failure (HF) are not well established. Moreover, the role of apoptosis in SHR is controversial. We investigated intracellular Ca^2+^, Ca^2+^-handling proteins and apoptosis in SHR vs. control Wistar rats (W) from 3 to 15 months (mo). Changes associated with the transition to HF (i.e. lung edema and decrease in midwall fractional shortening), occurred at 15 mo in 38% of SHR (SHRF). In SHRF, twitch and caffeine-induced Ca^2+^ transients, significantly decreased relative to 6/9 mo and 15 mo without HF signs. This decrease occurred in association with a decrease in the time constant of caffeine-Ca^2+^ transient decay and an increase in Na^+^/Ca^2+^ exchanger (NCX) abundance (p<0.05) with no changes in SERCA2a expression/activity. An increased Ca^2+^-calmodulin-kinase II activity, associated with an enhancement of apoptosis (TUNEL and Bax/Bcl2) was observed in SHR relative to W from 3 to 15 mo. Conclusions: 1. Apoptosis is an early and persistent event that may contribute to hypertrophic remodeling but would not participate in the contractile impairment of SHRF. 2. The increase in NCX expression/activity, associated with an increase in Ca^2+^ efflux from the cell, constitutes a primary alteration of Ca^2+^-handling proteins in the evolution to HF. 3. No changes in SERCA2a expression/activity are observed when HF signs become evident.

## Introduction

During chronic pressure overload, hypertrophy evolves to systolic left ventricular dysfunction and heart failure (HF). Experimental evidence suggests that a critical factor causing cardiac decompensation after pressure overload is the failure to preserve myocardial inotropy. Although the cause of cardiac dysfunction in HF is multifactorial, at the myocyte level two main mechanisms have been alleged to account for the decrease in myocardial contractility: 1. Alterations in Ca^2+^-handling and 2. Apoptosis. The first one is generally accepted as a signature feature of experimental and human HF [1], [2]. The second one is more controversial, although several studies indicate that low levels of chronically elevated myocyte apoptosis may play a causal role in the process that leads to HF [3]–[5].

The spontaneously hypertensive rat (SHR) is a genetic hypertension model widely used to study the natural evolution of hypertensive heart disease similar in many aspects to human essential hypertension. In this model, alterations in Ca^2+^ handling have been described at very early stages of the disease, even before the appearance of HF [6]. Surprisingly and in spite of the well-recognized deregulation of Ca^2+^ metabolism in SHR hearts, the mechanisms of Ca^2+^ handling alteration are not clear. Three main abnormalities that may account for Ca^2+^ mishandling in HF have been suggested: 1. A decrease in the expression of sarco-endoplasmic reticulum Ca^2+^ ATPase (SERCA2a) relative to its inhibitory protein phospholamban (PLN), which decreases sarcoplasmic reticulum (SR) Ca^2+^ reuptake. 2. An increased expression of Na^+^/Ca^2+^ exchanger (NCX), which enhances Ca^2+^ extrusion. 3. An hyperphosphorylation of the ryanodine receptor (RyR2), which renders the channel more prone to diastolic spontaneous Ca^2+^ release events (Ca^2+^ leak) [1], [2]. All three alterations converge to decrease the SR Ca^2+^ load.

Most reports studying the status of these proteins in SHR hearts are concentrated on a single one of them and/or covered a precise moment in the evolution of this disease. For instance, an increased NCX activity [7], [8] and either a decrease, increase or no change in SERCA2a expression/activity was described in young SHR [9]–[11]. At this stage, phosphorylation of PLN has been shown to be increased at the Ca^2+^-calmodulin-dependent kinase (CaMKII) site, Thr^17^ [9], [11], [12]. Moreover, in old SHR failing hearts either an increase in the abundance of NCX mRNA levels [13] or a decrease in NCX expression, attributed to a reduction in the t-tubular system [14], have been described. A decrease in SERCA2a expression [14] and a PKA-dependent hyperphosphorylation of RyR2 [15] were also observed at this later stage of the disease. In spite of the information that they provide, these approaches preclude a complete appraisal of the disease. This is mainly due to the fact that a given moment in the evolution of the illness results from a complex interplay between alterations causing cardiac disease and remodeling, tending to restore cardiac function. One possible strategy to gain insight into the mechanisms that lead to HF is to follow the disease progression over long time periods.

Apoptosis has also been described either as an early phenomenon [12] or as a final event in SHR hearts [16], [17]. However, the cells involved (myocytes/non-myocytes), the time course and the temporal association of the apoptotic phenomenon with the initiation of HF, still remain unsettled.

The main goal of the present study is to provide a sequential and mechanistic insight on the role of Ca^2+^-handling proteins and their impact on Ca^2+^ dynamics together with the degree of apoptosis, along the evolution to HF in SHR hearts. This strategy may offer a more realistic interpretation of the progression of the disease and will experimentally support new and rational therapeutic strategies.

## Materials and Methods

### Ethics Statement

All procedures were performed in accordance with the Guide for the Care and Use of Laboratory Animals [NIH Publication No. 85–23, revised 1996] and approved by the Ethics Committee of the Cardiovascular Research Center, National Research Council (CCT-La Plata Consejo Nacional de Investigaciones Científicas y Tecnológicas, Argentina) (P20-2010).

### Animals

Male SHR and age-matched normotensive Wistar rats (W) bred and maintained in our animal research facility were studied at 3, 6, 9, 15 months (mo) of age. Blood pressure was monitored weekly in non-anesthetized rats with the standard noninvasive tail cuff method (IITC, model 29-SSP). Before the animals were sacrificed, they were weighed and subjected to echocardiographic examination. The animals were then deeply anesthetized (35 mg/kg sodium pentobarbital IP) and after plane three of phase III of anesthesia was reached (verified by the loss of the corneal reflex and appearance of slow deep diaphragmatic breathing), heart and lungs were rapidly removed. Heart weight or lung weight-to-tibia length ratios were calculated. Hearts were assigned for biochemical studies, immunohistochemical staining (TUNEL assay), perfusion for contractile measurements and monophasic action potential determination or myocytes isolation for contractile and Ca^2+^ measurements. At 15 mo, a number of SHR showed HF clinical signs. The criterion to distinguish failing and non-failing animals was the presence of at least two clinical signs (labored breathing, tachypnea, cyanosis or diminished activity levels) and midwall fractional shortening ≤24.21% and lung weight/tibial length ≥7.71 mg/cm. 100, values which were two standard deviations below or above the mean of the SHR 15 mo group, respectively. The failing group was called SHRF and was considered separately.

### Echocardiographic Examination

Echocardiogram was performed in each rat under light anesthesia (inhalation of isoflurane at 1.5%). Cardiac geometry and function were evaluated by 2-dimensional M-mode echocardiography with a 7-MHz linear transducer (ATL G Model 11370 Ultramarc). All measurements, including left ventricular (LV) wall thickness and diastolic dimensions, were performed according to the American Society of Echocardiography method [18]. LV mass was calculated as previously described [19].

### Myocyte Isolation

Myocytes were isolated by enzymatic digestion [20] and kept in a HEPES buffered solution at room temperature (20–22°C), until used (see Methods in File S1).

### Intracellular Ca^2+^ Dynamics

Isolated myocytes were loaded with Fura-2/AM (10 µmol/L for 15 min). Intracellular Ca^2+^ was measured with an epi-fluorescence system (Ion Optix, Milton, MA). Briefly, dye-loaded cells were placed in a chamber on the stage of an inverted microscope (Nikon TE 2000-U) and continuously superfused with a HEPES buffered solution at a constant flow of 1 mL/min. Experiments were performed at room temperature (20–22°C) and myocytes were stimulated via two-platinum electrodes on either side of the bath at 0.5 Hz. By conducting experiments under these conditions, isolated myocytes are significantly less stressed, allowing for prolonged stimulation protocols and extended cell viability over time. Fura-2 fluorescence intensity was taken as an index of the intracellular Ca^2+^.

SR Ca^2+^ content was determined by rapidly switching to a HEPES buffered solution containing 25 mM caffeine to cause SR Ca^2+^ release. The rate constant of decay of caffeine-induced Ca^2+^ transient (kcaff) was used to estimate the velocity of Ca^2+^ extrusion by NCX (kNCX). kSERCA, was calculated by subtracting kcaff from the rate constant of decay of systolic Ca^2+^ transient (kt). These estimations assume that the decay of systolic Ca^2+^ transient is due to Ca^2+^ removal from the cytosol by both the SR and surface membrane mechanisms, mainly NCX, whereas the SR does not contribute to the decay of the caffeine response [21].

SR Ca^2+^ leak was studied according to Shannon et al. [22]. In short, the method consists of measuring resting Ca^2+^ in the presence and absence of SR Ca^2+^ channel blockade by 1 mmol/L tetracaine. After stabilization, to bring the cellular Ca^2+^ content to a steady state, the stimulation was stopped and the myocytes were exposed to 0 Na^+^-0 Ca^2+^ solution (Na^+^ replaced by choline chloride) for 30 s to block the NCX, so that little or no Ca^2+^ can enter or leave the resting cell. The difference in diastolic Ca^2+^ with and without tetracaine was taken as an estimation of SR Ca^2+^ leak.

Fluorescence data were stored for an off-line analysis (ION WIZARD fluorescence analysis software). Ca^2+^ transients were analyzed as the mean value over a 10–12 records for each cell.

### Heart Perfusion and Monophasic Action Potentials

Isolated hearts from SHR and W were perfused according to the Langendorff technique at constant temperature (37°C) and flow (14 ml) for 20 min, to evaluate contractility, relaxation and epicardial monophasic action potentials [23] (see Methods in File S1).

### Western Blot

Heart ventricles were freeze-clamped and pulverized. Briefly, 0.1 g of tissue was homogenized in 4 volumes of lysis buffer (in mmol/L: 30 KH_2_PO_4_, 25 NaF, 300 sucrose, 0.1 EDTA plus proteases inhibitor cocktail). Protein was measured by the Bradford method using BSA as standard. Lysates were separated per gel line in 10% SDS polyacrilamide gel [24] or 6% SDS polyacrilamide gel and transferred to polyvinylidene difluoride membranes. Blots were probed overnight with the following antibodies: Bcl-2 1∶1000 (Santa Cruz biotechnology, Santa Cruz, CA, USA), Bax 1∶1000 (Santa Cruz biotechnology, Santa Cruz, CA, USA), Sarcoplasmic Reticulum Ca^2+^-ATPase (SERCA2a) 1∶1000 (Thermo Scientific, Rockford, IL, USA), Na^+^/Ca^2+^ exchanger (NCX) 1∶1000 (Millipore, Billerica, MA, USA), phospholamban (PLN) 1∶1000 (ABR, Golden, CO, USA), Thr^17^ and Ser^16^-phosphorylated PLN 1∶1000 (pThr^17^ and pSer^16^, respectively) (1∶5000 (Badrilla, Leeds, UK), Ryanodine Receptors (RyR2) 1∶1000 (ABR, Golden, CO, USA), Ser^2814^ and Ser^2809^-phosphorylated RyR2 1∶1000 (Badrilla, Leeds, UK), pCaMKII 1∶1000 (Abcam, Cambridge, MA, USA). Apoptotic cell death was determined as the ratio of the signals between the pro-apoptotic Bax and the anti-apoptotic Bcl-2. GAPDH 1∶5000 (Millipore, Billerica, MA, USA) or calsequestrin (ABR, Golden, CO, USA) 1∶2000 signals were used to normalize the signal intensity of the different proteins. Immunoreactivity was visualized by a peroxidase-based chemiluminescence detection kit (Millipore, Billerica, MA, USA) using a Chemidoc Imaging system. The signal intensity of the immunoblot bands was quantified using Image J software (NIH).

### TUNEL Assay

TUNEL (In Situ Cell Death Detection Kit, TMR red, Roche, Mannheim, Germany) positive cells were imaged under a confocal microscope (Olympus FV-1000, Japan) using a 40x NA 0.95 objective and counted in 20 random fields for each experimental situation using the Olympus cellSens image analysis software v6.3. Results were expressed as percentage of TUNEL positive cells related to total number of cells. DAPI (1 ug/mL, 4, 6-Diamidino-2-phenylindole dihydrochloride, Sigma, St Louis, MO) was used for nuclear staining [25]. DIC (Differential Intereference Contrast) images helped to identify cardiomyocytes and non-cardiomyocyte cells. Cardiomyocytes were also identified following morphologic (nuclei with elliptical shape, striated cytoplasm) and morphometric parameters [26].

### Statistics

All data are presented as mean ± SEM. Comparisons within groups were made by unpaired Student’s t test, as appropriate. One-way ANOVA was used for multigroup comparisons. The Tukey post-hoc test was used to examine statistical differences observed with the ANOVA. A value of p<0.05 was taken to indicate statistical significance.

## Results

### Progressive Development of HF in SHR

**Figure 1:**
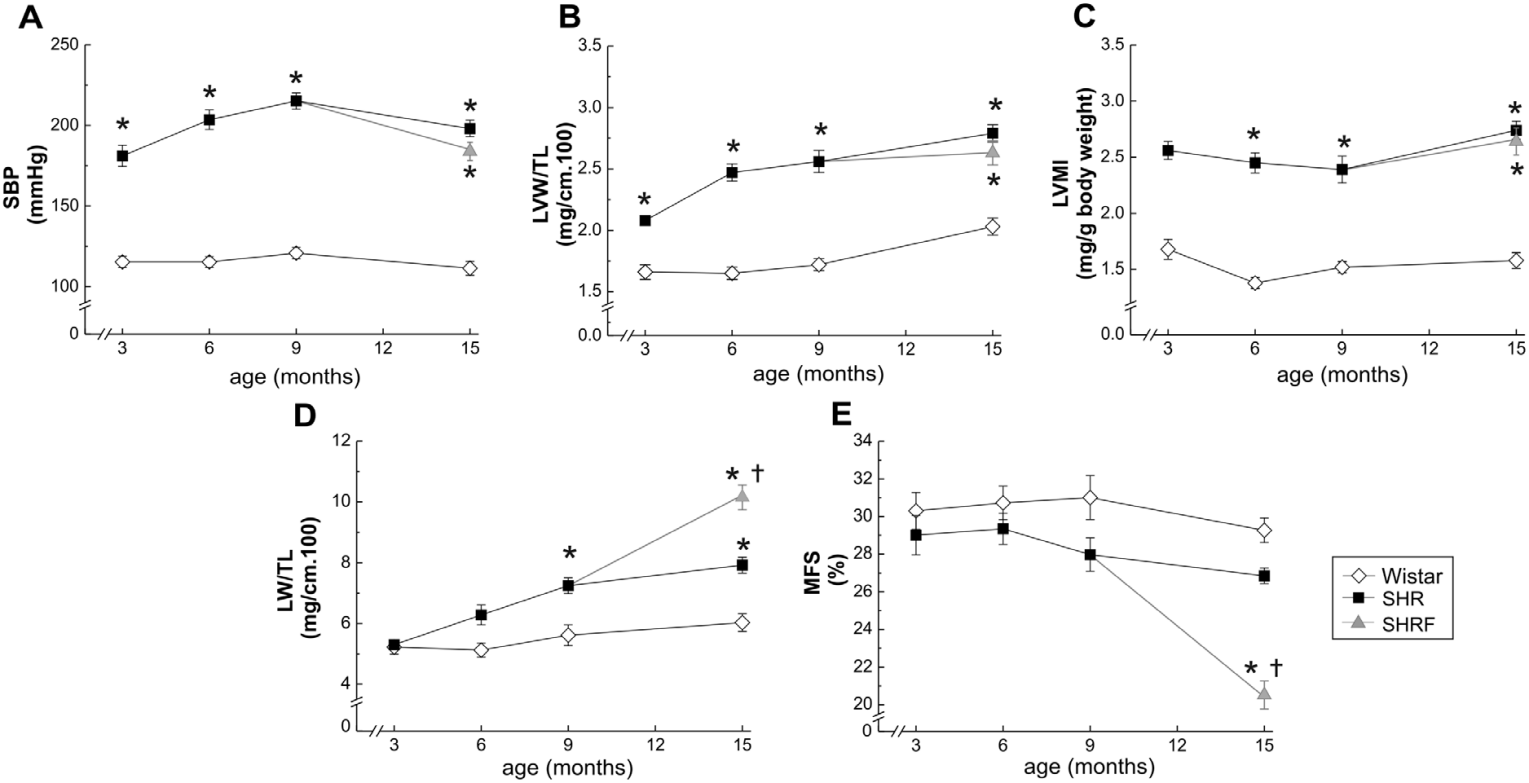
Progression from hypertrophy to heart failure in Wistar and SHR. A) Systolic blood pressure (SBP). B) Left ventricular wall/tibial length ratio (LVW/TL). C) Echocardiographic left ventricular mass index (LVMI). D) Lung weight-to-tibial length ratio (LW/TL). E) Left ventricle midwall fractional shortening (MFS). All the above mentioned parameters were evaluated in normotensive (Wistar), hypertensive (SHR) and hypertensive with significant signs of contractile failure (SHRF), at 3, 6, 9, 12 and 15 months of age. Hypertension and hypertrophy were evident from early stages in SHR (A, B and C) while heart failure signs appeared at late stages (D and E). In 15 mo SHR, the evolution of the disease allowed to define two groups with significantly different contractile impairment. *p<0.05 with respect to W of the same age; †p<0.05 with respect to SHR at 15 mo; n≥8 animals per point.

Hypertensive heart disease develops progressively in SHR from 3 to 15 mo. Systolic blood pressure and progression of hypertrophy (measured by left ventricular weight-to-tibial length and echocardiography), are shown in Figure 1A–C and Table S1 in File S1. This hypertrophic pattern has been generally considered an adaptive mechanism to increased afterload [27]. Signs of HF, i.e. increased lung weight-to-tibial length (LW/TL) and decreased midwall fractional shortening (MFS), became evident at 15 mo (Figure 1D and E) in 38% of rats (SHRF), in agreement with previous reports, where two groups of SHR were detected, failing and non-failing [16], [28]. As shown, whereas SHR at 15 mo did not further change LW/TL and MFS with respect to 9 mo, SHRF showed a highly significant increase and decrease in these parameters, respectively, as well as changes in left ventricle chamber dimensions at systole (Table S1 in File S1).

### Intracellular Ca^2+^ Dynamics in Isolated Myocytes

**Figure 2:**
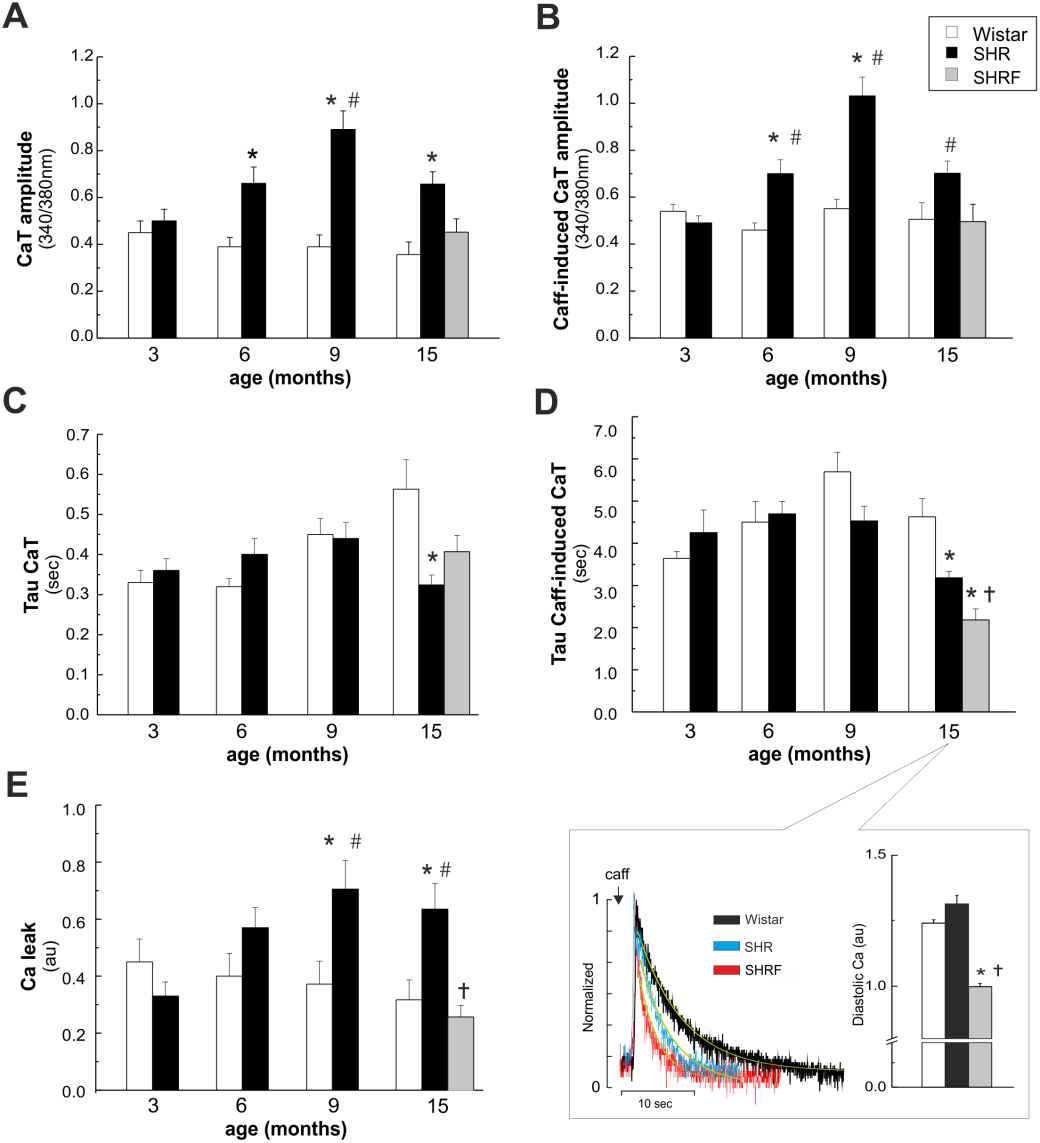
Intracellular Ca^2+^ dynamics in isolated myocytes of Wistar and SHR. A) Twitch Ca^2+^ transient (CaT) amplitude. B) Caffeine (Caff)-induced CaT amplitude. C) and D) Twitch and Caff-induced CaT decay parameters (tau). The inset in panel D) shows typical records of Caff-induced CaT (left) and diastolic Ca^2+^ (right) in W and SHR at 15 mo and in SHRF. E) Ca^2+^ leak from the SR. CaT amplitude and SR Ca^2+^ content (Caff-induced CaT) were higher in SHR at 6, 9 and 15 mo than in W of the same age. These increases were not observed in SHRF. At 15 mo there was a significant decrease in Tau of CaT and Caff-induced CaT in SHR relative to W. Tau of Caff-induced CaT in SHRF significantly diminished with respect to 15 mo SHR. At 9 and 15 mo there was an increase in SR Ca^2+^ leak in SHR vs. W, which did not occur in SHRF. *p<0.05 with respect to W of the same age; #p<0.05 with respect to SHR at 3 mo of age; †p<0.05 with respect to SHR 15 mo; n≥16 from at least 3 animals per group.

Figure 2A and B depicts overall results of intracellular twitch and caffeine-induced Ca^2+^ transient dynamics. At 6, 9 and 15 mo there was an increase in the amplitude of Ca^2+^ transient and SR Ca^2+^ content (caffeine-induced Ca^2+^ transient) in SHR vs. W consistent with previous findings [28]–[31]. These two parameters were also higher in SHR vs. 3 mo in the same strain and occur associated with the hypertrophic phenotype of SHR (Figure 1B and C). In contrast, in SHRF there were no significant differences in the amplitude of twitch and caffeine Ca^2+^ transients with respect to W myocytes, either relative to the same age or to SHR at 3 mo. This different pattern was due to a significant decrease in twitch and caffeine Ca^2+^ transient amplitude in SHRF myocytes with respect to the previous stage. At 15 mo there was a significant decrease in the time constant of caffeine-induced Ca^2+^ transient decline in SHR relative to W myocytes that was even more important in SHRF (Figure 2D and inset, left panel). The higher velocity of caffeine-induced Ca^2+^ transient decay indicates an increased NCX activity. This event may partially or totally account for the significant decrease in caffeine and twitch Ca^2+^ transient amplitude in SHR and SHRF compared to 9 mo. Up-regulation of NCX activity would be expected to promote a net loss of Ca^2+^ from the cell leading to decreased diastolic Ca^2+^. Indeed, diastolic Ca^2+^ was significantly reduced in SHRF with respect to 15 mo W, however it was not modified in the non-failing group (SHR) (Figure 2D inset, right panel). At 9 and 15 mo there was an increase in SR Ca^2+^ leak in SHR vs. W shown in Figure 2E. This increase could be associated with a significant increase in SR Ca^2+^ content observed in SHR at these stages (Figure 2B). The enhanced SR Ca^2+^ leak at 15 mo SHR could explain the lack of change in diastolic Ca^2+^ observed in this group vs. W of the same age. SR Ca^2+^ leak did not occur in SHRF and therefore it cannot represent a cause of diminished contractility in these failing rats.

**Figure 3:**
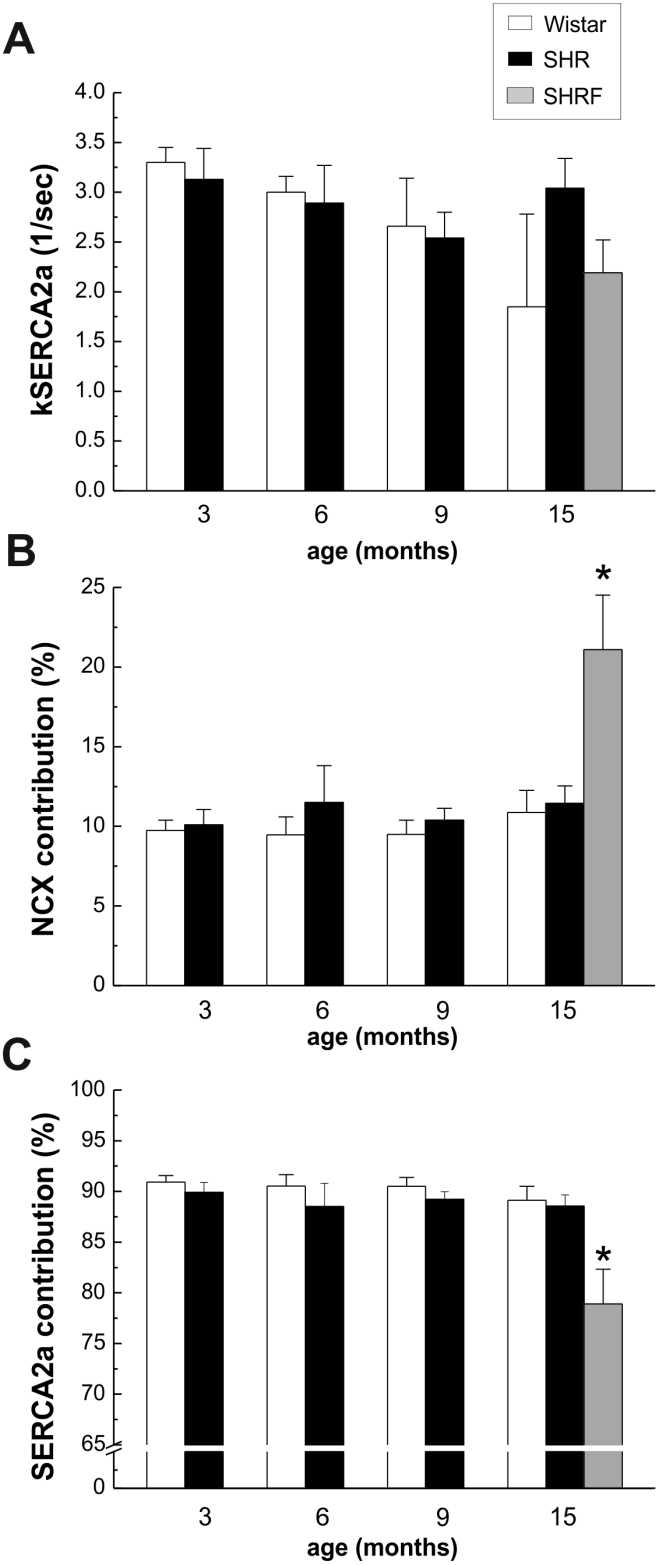
Activity of SERCA2a and relative contribution of NCX and SERCA2a to the Ca^2+^ transient constant. A) The estimated SERCA2a activity (kSERCA2a) did not show any difference between SHR and W myocytes at all the time points studied. B) The relative contribution of NCX to the Ca^2+^ transient decay constant was significantly increased in SHRF. C) The relative contribution of SERCA2a to the Ca^2+^ transient decay constant was significantly decreased in SHRF. *p<0.05 with respect to W of the same age.

Estimated activity of SERCA2a, (kSERCA2a), did not show any difference between SHR and W myocytes all along the ages studied (Figure 3A). However at 15 mo, SHRF show a significant increase and decrease in the relative contribution of NCX and SERCA2a respectively, in comparison to W and SHR (Figure 3B and C).

### Time Course of Expression and Phosphorylation of Different Proteins Involved in Intracellular Ca^2+^ Handling

**Figure 4:**
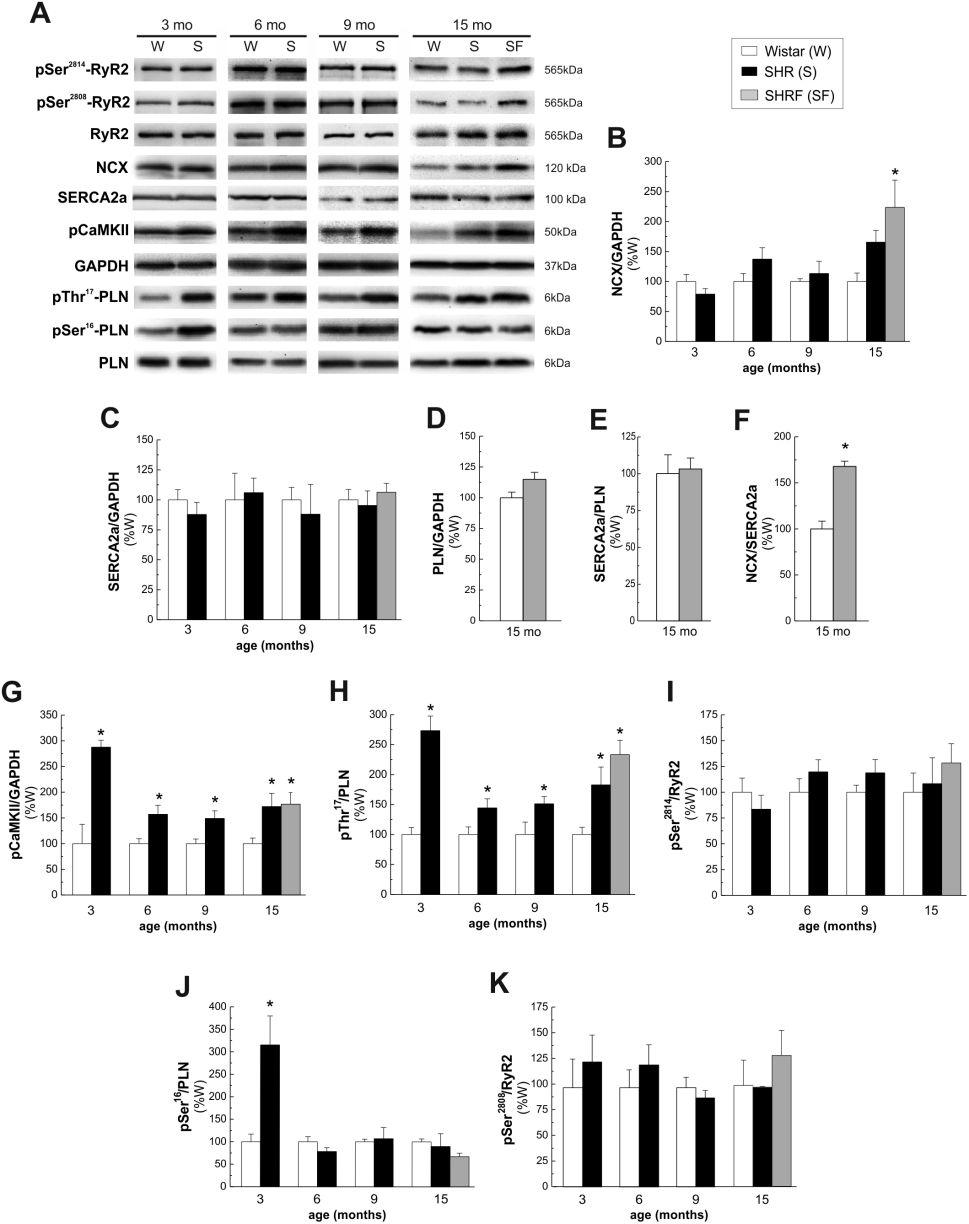
Time course of expression and phosphorylation of different proteins involved in intracellullar Ca^2+^ handling. A) Representative immunoblots and average results of the expression and/or phosphorylation of B) NCX; C) SERCA2a; D) PLN; E) SERCA2a/PLN ratio; F) NCX/SERCA2a ratio; G) pCaMKII; H) pThr^17^-PLN; I) pSer^2814^-RyR2; J) pSer^16^-PLN; K) pSer^2808^-RyR2. The results are expressed as percentage of values obtained in W of the same age. Protein levels were normalized to the loading control glyceraldehyde 3-phosphate dehydrogenase (GAPDH). Phosphorylation of PLN and RyR2 was expressed as ratio between phosphorylated and non-phosphorylated forms of the proteins. While SERCA2a expression showed no differences at any time point studied, the expression of NCX was significantly higher only in SHRF. In this latter group, PLN expression and SERCA2a/PLN ratio did not change with respect to W, therefore the ratio NCX/SERCA2a was significantly enhanced. CaMKII and Thr^17^-PLN phosphorylations significantly increased from 3 mo in SHR with respect to W. PKA-dependent Ser^16^ phosphorylation of PLN increased at 3 mo and then decreased. Phosphorylation of Ser^2808^ and Ser^2814^ of RyR2 did not change at any age studied. *p<0.05 with respect to W of the same age; n≥4 animals per group.

Figure 4 shows immunoblots (A) and overall results (B–K) of the time course of the expression and phosphorylation of different proteins involved in excitation-contraction coupling in cardiomyocytes. The expression of NCX tended to increase at 15 mo (SHR) and reached significant levels with respect to W, only in animals that developed HF signs (SHRF) (Figure 4B). Our results support a tight association between NCX overexpression (Figure 4B) and the acceleration in the caffeine Ca^2+^ transient decay (Figure 2D), which could lead, at least in part, to the decrease in the amplitude of twitch and caffeine Ca^2+^ transients at the cellular level (Figure 2A and B) and the appearance of HF signs in the intact animal (Figure 1D and E).

Although a decrease in SERCA2a has been described as a hallmark in cardiac failure, we could not find any difference in either SERCA2a, PLN expression, the SERCA2a/PLN ratio (Figure 4C, D and E) or the estimated activity of SERCA2a in SHRF (Figure 3A) when compared to W of the same age. From these results it is evident that a decrease in SERCA2a expression/activity is not necessary or sufficient by itself, for the initiation of cardiac impairment [32]. Instead, the enhancement of the ratio NCX/SERCA2a (Figure 4F) due, in our model, to an increase in NCX expression/activity in SHRF and the consequent Ca^2+^ efflux from the cell, seems to be a requirement to decrease SR Ca^2+^ content with respect to previous stages and a primary event in the impairment of heart function at this stage.

CaMKII and Thr^17^-PLN phosphorylations significantly increased from 3 mo (Figure 4G and H). In contrast, the CaMKII site of RyR2, Ser^2814^, did not change significantly (Figure 4I). PKA-dependent Ser^16^ phosphorylation of PLN increased at 3 mo and then decreased (Figure 4J) whereas Ser^2808^ of RyR2, a predominant PKA site [33], did not change in the evolution from hypertrophy to HF (Figure 4K). The dual phosphorylation of PLN at 3 mo (Ser^16^ and Thr^17^ sites), was not associated with any significant increase in the rate of Ca^2+^ transient decay (Figure 2C). Experiments in perfused hearts from SHR and W of 3 mo also confirmed a significant increase in PLN phosphorylation in SHR hearts which was not associated with a relaxant effect. In these experiments a significant prolongation of the action potential was observed, a mechanism which could be offsetting the relaxant effect of PLN phosphorylation (Figure S1 in File S1).

### Apoptosis: An Early Event in SHR Hearts

**Figure 5:**
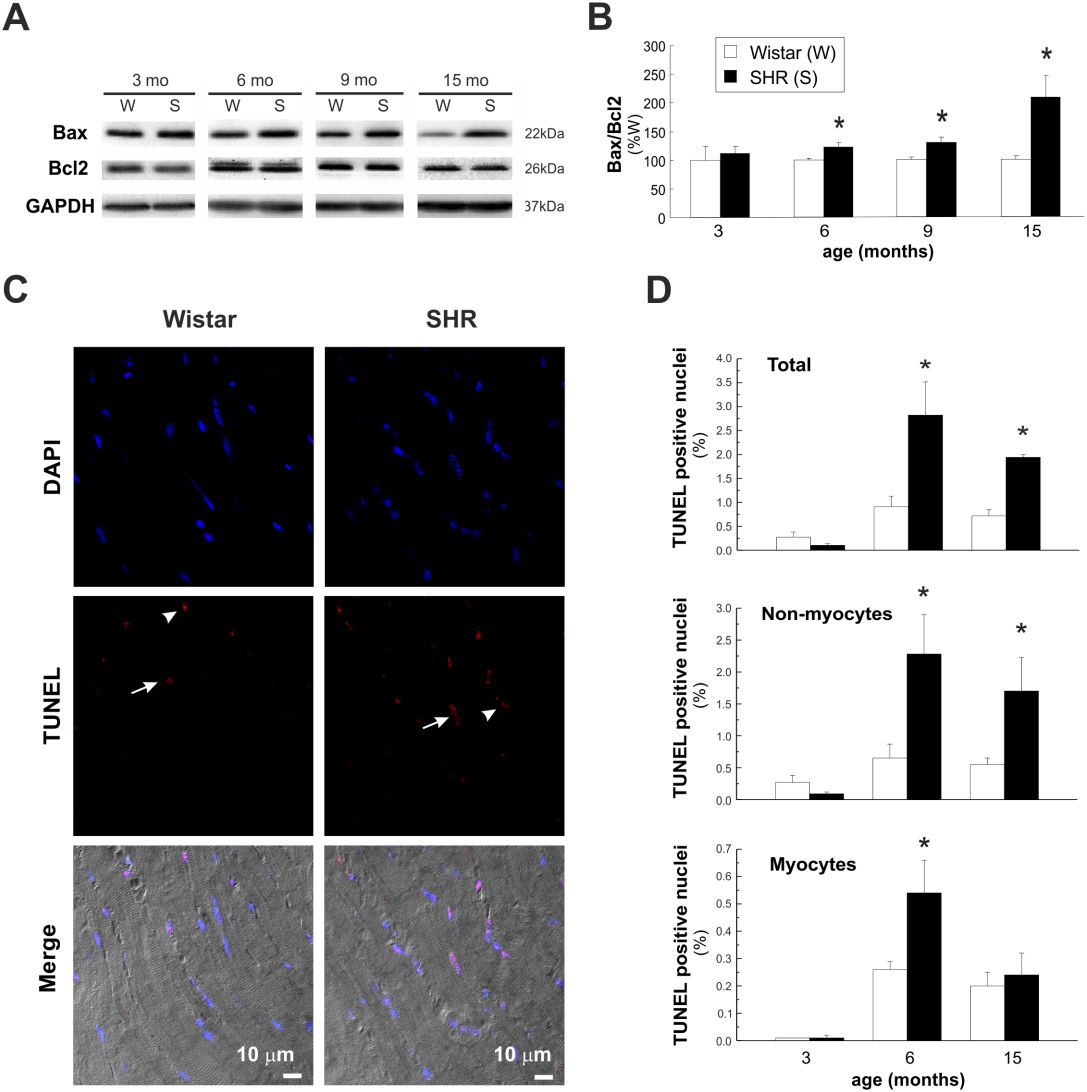
Apoptosis in Wistar and SHR hearts. A) Representative blots showing the expression of the proapototic (Bax) and antiapoptic (Bcl2) proteins in Wistar (W) and SHR (S) hearts at different ages. B) Quantitative analysis of protein expression, indicate increased Bax/Bcl2 ratio in SHR hearts at 6, 9 and 15 mo of age. (n≥4 animals per group). C) Typical example of TUNEL assay of 6 mo W and SHR and D) overall results of these experiments (n≥5 animals per group). Arrows indicate myocytes and arrow heads non-myocytes. Apoptosis is an early event in SHR hearts due to myocytes and non-myocytes death at 6 mo and to non-myocytes cells death at 15 mo. *p<0.05 with respect to W of the same age.

Figure 5A and B shows western blots and overall results of the ratio between the proapoptotic and antiapoptotic proteins, Bax and Bcl2, respectively. From 6 mo, this ratio significantly increased in SHR with respect to W. To further explore apoptosis, we performed TUNEL assay at 3, and 15 mo. Of note, since we did not detect any significant difference between values of Bax/Bcl2 ratios in SHR and SHRF at 15 mo, both groups were considered as a single one. This finding indicates that apoptosis can hardly contribute to HF signs in SHR. Figure 5C and D compares a typical example and overall results of TUNEL assay in W and SHR. At 6 and 15 mo there was a significant increase in total TUNEL positive nuclei relative to W. However, whereas at 6 mo this increase was due to both, myocyte and non-myocyte cells, at 15 mo, it was mainly due to a significant increase in non-myocyte cells (Figure 5D). Non-myocytic death was significantly more important than myocyte death, both in SHR and W hearts (note the different scales).

Taken together, the results indicated that apoptosis is an early event in SHR that might influence hypertrophic remodeling in these hearts. Apoptotic cell death persists all along the evolution from hypertrophy to HF although the relative contribution of myocytes and non-myocytes varied with the progression of the disease.

## Discussion

The present results depict the sequential changes of Ca^2+^ handling, Ca^2+^ handling proteins and cell death, three major factors thought to be involved in the decreased contractility during the progression from hypertrophy to HF in SHR. The data reveal that the increased expression of NCX appears as a point of inflection in the evolution to HF in this model.

The main outcomes of this study are the following: 1. An enhancement of CaMKII activity is the first detected molecular event associated to hypertrophy that persists to the HF stage. 2. An early rise of myocyte apoptosis (6 and 9 mo) which, although with a different cellular pattern, also remains until pump failure was reached. 3. An increased expression/activity of NCX, which might underlie, at least in part, the Ca^2+^ mishandling and contractile deterioration of failing hearts. 4. A lack of decrease in SERCA2a expression/activity when HF signs appeared.

The results obtained highlight the fact that there is a continuum of different and transitory alterations in Ca^2+^ dynamics, Ca^2+^ handling proteins and cell death in the evolution from hypertrophy to HF. These findings may help to explain the diversity of results obtained when these variables are studied at a given time in the course of the disease. In addition, our results allow us to emphasize that apoptosis and decreased SERCA2a are not sufficient to induce heart function impairment in the SHR model. On the other hand, the increased NCX expression/activity, and the consequent unbalanced NCX/SERCA2a, seems to be a main trigger event in the progression to HF.

### Apoptosis and Increased CaMKII Activity: Two Early Events in the Evolution to HF

It has been shown that very low levels of myocyte apoptosis are observed in patients with dilated cardiomyopathy [34]. However, other studies were unable to show apoptosis in the progression to human HF [4]. The occurrence of apoptosis is also controversial in SHR hearts. Whereas we recently showed apoptotic events at an early stage [12], Zhao et al. [35] found a decrease in apoptotic cells in hearts from SHR of a similar age. Other studies concluded that apoptosis either increased or was not responsible for HF in SHR [16], [17]. Moreover the cell type in which apoptosis occurs (myocytes [16] and/or non-myocytes [36]) is also under discussion. One of the reasons of these disparate results may lie in the fact that these previous studies assessed different single time points in the evolution to HF. Our results showed that apoptosis is a continuous and significant event from 6 mo old SHR, confirming that it is an early process in hypertensive disease. Furthermore they suggest that apoptosis may contribute to the hypertrophic heart remodeling that precedes pump failure. The mixed apoptosis (myocytes and non-myocytes) described in this work, evolved to an exclusively non-myocyte apoptosis in the advanced stages of the illness. The cause and consequences of this interesting finding is out of the scope of the present study and deserves further investigation.

In addition, our results show an increase in CaMKII activity from 3 mo of age in SHR which persists all along the evolution from hypertrophy to HF. This increase precedes the onset of apoptotic cell death, reinforcing the idea of a causal relationship between both events, as previously described [12], [37], [38]. Moreover, recent experiments indicated that the increased activity of CaMKII upregulates NCX expression [39]. Thus, the increase in CaMKII observed in the present study might be underlying the enhancement of both, apoptosis and NCX expression.

### Role of NCX in the Evolution of Hypertrophy to HF

A main finding of this study is the significant enhancement in the estimated Ca^2+^ efflux through an overexpressed NCX at 15 mo in SHRF. The activity of NCX depends not only on the kinetics properties or expression level, but also on the magnitude and change of the driving force. The driving force is a function of intracellular sodium ([Na^+^]_i_), [Ca^2+^]_i_ and transmembrane potential. In HF, the commonly found increased [Na^+^]_i_, prolongation of the action potential and altered [Ca^2+^]_i_, favor the ratio reverse mode/forward mode of NCX, decreasing Ca^2+^ efflux [40]. Despite this scenario, an increased Ca^2+^ efflux was detected in the current experiments in SHRF which should be secondary to the higher exchanger expression and could explain, at least in part, the decrease in SR Ca^2+^ load and Ca^2+^ transient amplitude in the isolated myocytes. Moreover, the finding that no significant changes in NCX expression were observed in 15 mo SHR that did not present HF signs, further suggests the involvement of increased NCX expression in HF progression.

Moreover the increase in NCX expression appears as an independent phenomenon and not as a compensatory mechanism tending to offset the negative effects on relaxation produced by the decrease in SERCA2a activity described in several HF models. This conclusion is in consonance with previous results in failing human myocardium, suggesting that regulation of the expression of both Ca^2+^ transporters occurs by different independent signals [41]. Indeed, two different phenotypes at the end stage of HF, one with increased NCX and unchanged levels of SERCA2a and a second one with markedly decreased SERCA2a expression and unchanged NCX were found. Interestingly, the ratio of NCX/SERCA2a was similar in both groups but considerably increased compared with non-failing human heart [41]. In agreement with this previous work, the results presented here showed that the ratio NCX/SERCA2a was only increased at 15 mo in SHRF where HF signs were evident.

Of note, overexpression of NCX in transgenic mice, leads to abnormal Ca^2+^ handling and is responsible for a decompensatory transition to HF under stress conditions [42], in full agreement with the present findings.

A decreased expression of SERCA2a has been noted in some experimental models of HF and in the failing human heart [43], [44]. Indeed, the benefits of SERCA2a adeno and adeno-associated viral transduction in humans and in different HF models are well documented [45], [46]. However, the present results demonstrated that the decrease in SERCA2a expression and in the estimated SERCA2a activity, is not a necessary phenomenon for the occurrence of cardiac dysfunction in SHR.

### PLN and RyR2 Phosphorylation in the Evolution to HF

In the present experiments a sustained increase in the phosphorylation of Thr^17^ site of PLN occurred from 3 mo and persisted all along the evolution of the disease. This phosphorylation may have contributed to the enhancement of SR Ca^2+^ load observed in isolated myocytes in SHR at 6–15 mo. However it was not associated to any evident relaxant effect as should be expected. A possible explanation for this finding is the prolongation of the action potential that is known to occur in SHR [6] and that we confirmed in the present results. This prolongation may offset the increase in the rate of Ca^2+^ transient decay that should have produced the phosphorylation of PLN. The lack of significant increase in SR Ca^2+^ load and Ca^2+^ transient or the very modest raise in contractility observed in 3 mo SHR myocytes and intact hearts, is more difficult to explain, although it is not a novel finding. Experiments by Boknik et al. [11] showed a significant increase in PLN phosphorylation associated to a decreased contractility in young SHR compared with normotensive animals.

The increase in Ser^16^ phosphorylation observed at 3 mo, vanished at 6–15 mo consistent with an early increased sympathetic tone followed by a downregulation of β-adrenergic system, during the course of the hypertensive disease [47].

Finally we did not find any significant change in the phosphorylation of Ser^2808^ and Ser^2814^ sites of RyR2. These results are at odds with previous reports which described an increase in Ser^2808^ and Ser^2814^-RyR2 phosphorylation in different HF models [48], [49]. It is possible that a slight difference in the balance between the status of phosphatases, that are known to be increased in HF [50], [51], and the activity of kinases, may explain the different results. In our model, RyR2 phosphorylation appears as a mechanism not involved in the systolic dysfunction at the failing stage and the increased SR Ca^2+^ leak at earlier ages can be fully explained by the high SR Ca^2+^ content.

In summary, although we are aware of the fact that an increase in NCX has been previously described in HF, these previous studies, particularly in SHR hearts, addressed a single moment in HF evolution and failed to show a causal relationship between the increase in NCX and the impaired contractility. To the best of our knowledge, this is the first study describing the sequence of the changes of three main factors that are critical in determining the contractile state of the heart, namely, Ca^2+^ handling, Ca^2+^ handling proteins and cell death, during the progression to HF. These results point to the enhanced expression/activity of the NCX and the consequent increase in the NCX/SERCA2a ratio, as an important mechanism that may contribute to the hypocontractile state typical of HF.

## Supporting Information

File S1 **File includes Table S1 and Figure S1.** Table S1. Echocardiographic parameters of the different experimental groups. Left ventricular end diastolic diameter (LVEDD); Left ventricular end systolic diameter (LVESD); Interventricular septum in systole (IVSs); Interventricular septum in diastole (IVSd); Posterior wall diastolic thickness (PWdt); Posterior wall systolic thickness (PWst). Progression of hypertrophy was observed from 3 mo SHR in IVSs, IVSd, PWdt and PWst. Moreover, 15 mo SHRF showed a highly significant increase in LVESD and IVSD. Values are expressed as mean ± SE. *p<0.05 vs. W. # p<0.05 vs SHR 15 mo. Figure S1. Ex vivo experiments: perfused hearts from Wistar and SHR at 3 mo of age. A) Representative recordings of left ventricular developed pressure (LVDP) and monophasic action potential (MAPs) simultaneously obtained in Langendorff perfused hearts. B) and C) overall results of half relaxation time (t1/2) and monophasic action potential duration at 90% repolarization (MAPD90). D) and E) typical immunoblots and overall results of the phosphorylation of PLN at Ser^16^ and Thr^17^ respectively. *p<0.05 with respect to Wistar hearts, n≥3 animals per group.
(DOCX)
