# Supplementary material for: Increased Na+/Ca2+ Exchanger Expression/Activity Constitutes a Point of Inflection in the Progression to Heart Failure of Hypertensive Rats
Source: PLoS One. 2014 Apr 29;9(4):e96400. doi: 10.1371/journal.pone.0096400 (PMC4004550; doi:10.1371/journal.pone.0096400)
Supplement: File S1 — File includes Table S1 and Figure S1. Table S1. Echocardiographic parameters of the different experimental groups. Left ventricular end diastolic diameter (LVEDD); Left ventricular end systolic diameter (LVESD); Interventricular septum in systole (IVSs); Interventricular septum in diastole (IVSd); Posterior wall diastolic thickness (PWdt); Posterior wall systolic thickness (PWst). Progression of hypertrophy was observed from 3 mo SHR in IVSs, IVSd, PWdt and PWst. Moreover, 15 mo SHRF showed a highly significant increase in LVESD and IVSD. Values are expressed as mean ± SE. *p<0.05 vs. W. # p<0.05 vs SHR 15 mo. Figure S1. Ex vivo experiments: perfused hearts from Wistar and SHR at 3 mo of age. A) Representative recordings of left ventricular developed pressure (LVDP) and monophasic action potential (MAPs) simultaneously obtained in Langendorff perfused hearts. B) and C) overall results of half relaxation time (t1/2) and monophasic action potential duration at 90% repolarization (MAPD90). D) and E) typical immunoblots and overall results of the phosphorylation of PLN at Ser16 and Thr17 respectively. *p<0.05 with respect to Wistar hearts, n≥3 animals per group. (DOCX) [file pone.0096400.s001.docx]

**Supporting information**

***File S1***

**Methods**

***Myocyte isolation***

Rat hearts were attached via the aorta to a cannula, excised and mounted in a Langendorff apparatus. They were then retrogradly perfused at 37 °C at a constant perfusion pressure of 70–80 mmHg with HEPES solution of the following composition (mM): 146.2 NaCl, 4.7 KCl, 1 CaCl_2_, 10.0 HEPES, 0.35 NaH_2_PO_4_, 1.05 MgSO_4_, 10.0 glucose (pH adjusted to 7.4 with NaOH). The solution was continuously bubbled with 100 % O_2_. After a stabilization period of 4 min, the perfusion was switched to a nominally Ca^2+^-free HEPES solution for 5 min. Hearts were then recirculated with collagenase (180 U/mL), 0.1 mg/mL pronase and 1 % bovine serum albumin (BSA), in HEPES containing 50 μM CaCl_2_. Perfusion continued until hearts became flaccid (15–25 min). Hearts were then removed from the perfusion apparatus by cutting at the atrio-ventricular junction. The isolated myocytes were separated from the undigested tissue and rinsed several times with a HEPES solution containing 1 % BSA and 500 μM CaCl_2_. After each wash, myocytes were left for sedimentation for 10 min. Myocytes were kept in HEPES solution at room temperature (20–22°C) until use. Rod-shaped myocytes with clear and distinct striations and obvious marked shortening and relaxation on stimulation were used. Cells that were unstable at 0.5Hz, that did not show an obvious positive inotropic effect in response to an increase in stimulation frequency, or that showed early signs of deterioration, such as large decreases in resting cell length at low stimulation frequencies, were discarded. Experiments were performed at room temperature.

***Heart Perfusion and monophasic action potentials***

Isolated rat hearts were perfused according to Langendorff technique at constant temperature (37°C), flow (14 ml/min) and heart rate (240 beats/min). The composition of the bicarbonate buffer solution was (in mmol/L) 128.3 NaCl, 4.7 KCl, 1.35 CaCl_2_, 20.2 NaHCO_3_, 0.4 NaH_2_PO_4_, 1.1 MgCl_2_, 11.1 glucose, and 0.04 Na_2_EDTA; equilibrated with 95% O2-5% CO2 to give a pH of 7.4. Mechanical parameters were obtained by passing into the left ventricle a latex balloon connected to a pressure transducer. The balloon was filled with aqueous solution to achieve a left ventricular end-diastolic pressure of 6–12 mmHg (1). Monophasic action potentials (MAPs) were obtained by using a Ag/AgCl electrode apposed to the epicardial free left ventricular wall, using a direct current-coupled high-input impedance differential amplifier. The MAP electrode was gradually positioned with the help of a micromanipulator until a gentle but stable contact pressure was achieved (2, 3). Recordings were accepted for analysis if they had a stable baseline, a rapid upstroke with consistent amplitude, and a smooth contoured repolarization phase and if they remained stable throughout the stabilization period.

REFERENCES

1. Mundiña-Weilenmann C, Ferrero P, Said M, Vittone L, Kranias EG, Mattiazzi A. Role of phosphorylation of Thr^17^ residue of phospholamban in mechanical recovery during hypercapnic acidosis. Cardiovasc Res. 66:114-122, 2005.
2. Bethell HW, Vanderberg JI, Smith GA, Grace AA. Changes in ventricular repolarization during acidosis and low-flow ischemia. Am J Physiol Heart Circ Physiol 275: H551–H561, 1998.
3. Knollmann BC, Katchman AN, Franz MR. Monophasic action potential recordings from intact mouse heart: validation, regional heterogeneity, and relation to refractoriness. J Cardiovasc Electrophysiol. 12:1286-1294, 2001.

**Table S1. Echocardiographic parameters of the different experimental groups.**

|  | **3 mo** | | **6 mo** | | **9 mo** | | **15 mo** | | |
| --- | --- | --- | --- | --- | --- | --- | --- | --- | --- |
|  | **W** | **SHR** | **W** | **SHR** | **W** | **SHR** | **W** | **SHR** | **SHRF** |
|  | (n=17) | (n=16) | (n=12) | (n=13) | (n=11) | (n=12) | (n=22) | (n=25) | (n=15) |
| **LVEDD** (mm) | 5.82±0.16 | 5.92±0.09 | 6.03±0.10 | 6.36±0.13 | 6.30±0.15 | 6.26±0.15 | 6.36±0.11 | 6.62±0.11 | 6.47±0.12 |
| **LVESD** (mm) | 2.23±0.10 | 2.16±0.08 | 2.28±0.08 | 2.30±0.09 | 2.42±0.15 | 2.32±0.09 | 2.46±0.06 | 2.67±0.06 | 2.91±0.10*# |
| **IVSs** (mm) | 1.49±0.04 | 1.76±0.07* | 1.60±0.03 | 1.87±0.02* | 1.58±0.03 | 1.93±0.03* | 1.65±0.03 | 1.97±0.03* | 1.92±0.03* |
| **IVSd** (mm) | 2.90±0.08 | 3.35±0.09* | 2.98±0.07 | 3.61±0.11* | 2.98±0.07 | 3.43±0.10* | 3.24±0.07 | 3.65±0.07* | 3.93±0.10*# |
| **PWdt** (mm) | 1.53±0.04 | 1.87±0.07* | 1.59±0.03 | 2.01±0.06* | 1.67±0.06 | 2.08±0.06* | 1.66±0.04 | 2.04±0.04* | 2.06±0.05* |
| **PWst** (mm) | 2.87±0.09 | 3.30±0.08* | 3.03±0.09 | 3.52±0.06* | 3.11±0.07 | 3.85±0.14* | 3.17±0.06 | 3.63±0.08* | 3.69±0.11* |

Left ventricular end diastolic diameter (LVEDD); Left ventricular end systolic diameter (LVESD); Interventricular septum in systole (IVSs); Interventricular septum in diastole (IVSd); Posterior wall diastolic thickness (PWdt); Posterior wall systolic thickness (PWst). Progression of hypertrophy was observed from 3 mo SHR in IVSs, IVSd, PWdt and PWst. Moreover, 15 mo SHRF showed a highly significant increase in LVESD and IVSD. Values are expressed as mean ± SE. * p<0.05 vs. W. # p<0.05 vs SHR 15 mo.

***
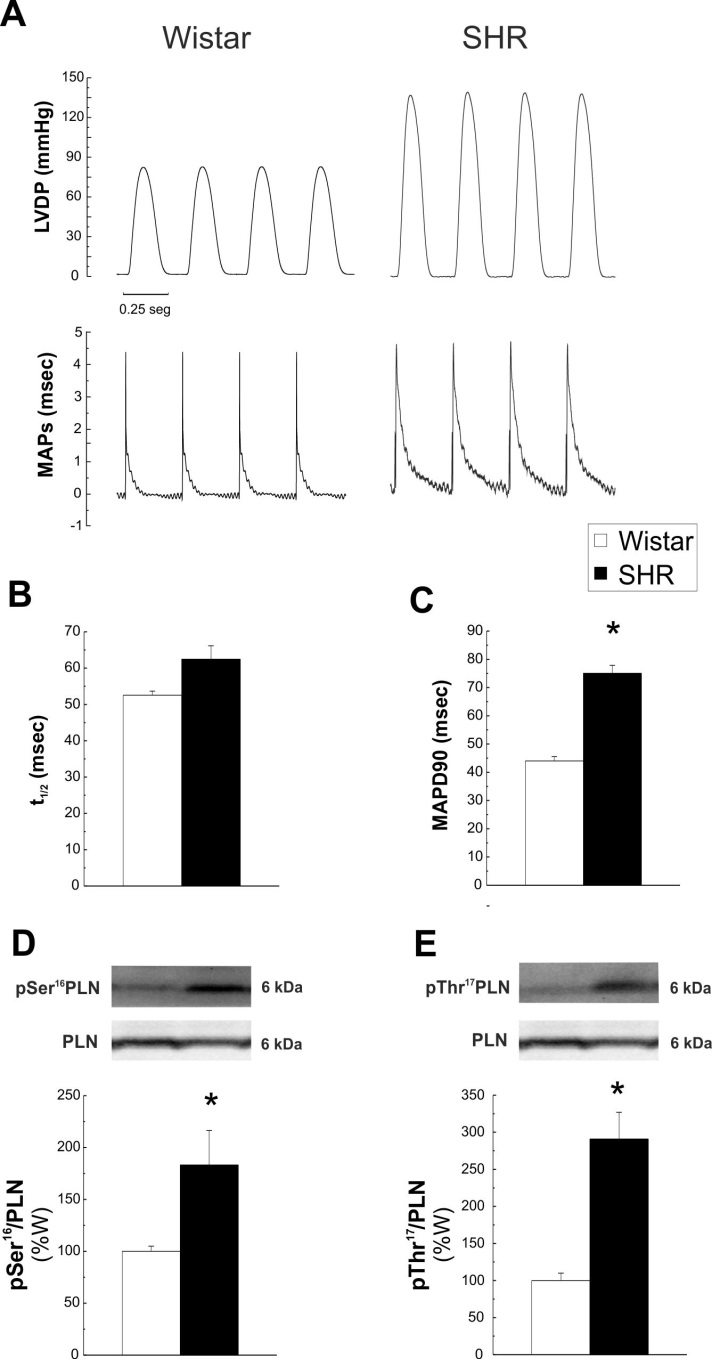
***

**Figure S1. Ex vivo experiments: perfused hearts from Wistar and SHR at 3 mo of age.** A) Representative recordings of left ventricular developed pressure (LVDP) and monophasic action potential (MAPs) simultaneously obtained in Langendorff perfused hearts. B) and C) overall results of half relaxation time (t1/2) and monophasic action potential duration at 90% repolarization (MAPD90). D) and E) typical immunoblots and overall results of the phosphorylation of PLN at Ser^16^ and Thr^17^ respectively. * p<0.05 with respect to Wistar hearts, n≥3 animals per group.
